# Repeated exposure of acidic beverages on esthetic restorative materials: An *in-vitro* surface microhardness study

**DOI:** 10.4317/jced.52906

**Published:** 2016-07-01

**Authors:** Arun M. Xavier, Steffy M. Sunny, Kavita Rai, Amitha M. Hegde

**Affiliations:** 1 Reader. Department of Pediatric dentistry, Amrita School of Dentistry, Amrita Vishwa Vidyapeetham, Cochin - 41, India; 2 Chief Dental Surgeon. Smile n Care Multispeciality & Pediatric Dental Home, Aluva, Kerala, India; 3 Professor & Head of Department. Dept of Pedodontics and Preventive Dentistry, Bangalore Institute of Dental Sciences, Wilson Garden, Hosur main road, Lakksandra, Bangalore, 560029, India; 4 Senior Professor & Head. Department of Pedodontics and Preventive Children Dentistry, A.B. Shetty Memorial Institute of Dental Sciences, Mangalore 18, India

## Abstract

**Background:**

A manifold increase in the consumption of aerated beverages has witnessed a twin increase in tooth wear and raised demand for esthetic restorative materials. This study aimed to evaluate the surface microhardness changes of esthetic restorative materials following treatment with aerated beverages in an *in-vitro* situation.

**Material and Methods:**

The initial surface microhardness of the restorative materials GC Fuji II LC, GC Fuji IX, Nano Glass ionomer, Resin and Nano composite was recorded. These materials were studied under 3 groups that included those exposed to the acidic beverages daily, weekly once in a month and those that had no exposures at all. The final surface microhardness of the materials was recorded following experimentation and was subjected to statistical comparisons.

**Results:**

The restorative materials were compared for their surface microhardness changes following respective treatments using the T-test and One-way ANOVA analysis. Inter-comparisons between the groups showed statistical significance (*p*<.05), when treated with both the beverages. The five restorative materials revealed surface microhardness loss; the maximum reduction noticed with the Nano glass ionomer cement tested (*p*<.0005).

**Conclusions:**

The surface microhardness of restorative materials markedly reduced upon repeated exposures with acidic beverages; the product with phosphoric acid producing the maximum surface microhardness loss.

** Key words:**Restorative materials, acidic beverages, surface microhardness, resin composites, glass ionomers.

## Introduction

Excessive consumption of acidic food and beverages, or unusual eating and drinking habits such as sipping an acidic drink over a long period of time have proven to increase the acid challenge to teeth (1). However, the extent of impact of acidic interactions in the oral environment is not yet conclusively established.

Esthetic restorative materials are marketed in various types with different physical characteristics and colors. However, under acidic conditions, all dental restorative materials have shown degradation over time (2). Deterioration at low pH, low resistance to wear and high technique sensitivity are few reported drawbacks of glass ionomer cements. Studies on organic acids of plaque and influence of food-simulating solvents in oral cavity have shown a critical influence on *in-vivo* degradation of composite resins and glass ionomer cements (3). A study based on Coca-Cola has shown higher percentage surface roughness on glazed as well as polished surfaces of low-fusing ceramic and All-ceramic materials, when compared to acidic solutions of APF gel and bleaching agents like carbamide peroxide (4).

As hardness is related to materials strength, proportional limit and its ability to abrade by opposing dental structures/materials (5), any chemical softening from beverages of low pH might have implications on the clinical durability of restorations. Due to the rise in consumption of aerated beverages and wider usage of tooth-colored restorative materials, an experimental based approach is inevitable to reveal the surface morphological changes of such dental materials following exposures to beverages of low pH. This study thus aimed to assess the surface microhardness changes of various esthetic restorative materials upon repeated exposure to acidic beverages in an *in-vitro* trial.

## Material and Methods

-Restorative specimens & Beverages

Five esthetic restorative materials GC Fuji II LC Improved (A2, GC Corporation, Tokyo, Japan), Fuji IX (GC High Strength Posterior Restorative, GC Corporation, Tokyo, Japan), KetacTM N100 (3M ESPE, USA), Filtek Z350 universal restorative Composite (3M ESPE, USA) and Ceram XTM Nano ceramic restorative (Dentsply, Mono M6 = A3.5, B3, B4) were included in this study. The sample size was computed based on a previous study by Hengtrakool *et al.*, which studied the effect of naturally acidic agents on the microhardness and surface micromorphology of restorative materials (6). With a 90% power and 95% confidence interval, the minimum sample size was statistically derived as 5. However, the current investigation considered a total of 10 samples per group.

The materials were manipulated according to manufacturers’ instructions and placed in rectangular recesses (3 mm wide, 4 mm long and 2 mm deep) of customized acrylic moulds. Light cure composites were polymerized with a curing light (Spectrum; Dentsply Inc. Milford, DE 19960) over a glass slide (7). Chemical-cure materials were left to set at room temperature for 10 mins. After light polymerization/setting, the materials were removed from the customized molds.

The aerated beverages used for testing the specimens included two Coca Cola products having pH of 2.5 and 2.98 respectively, measured using a pH meter (Hanna Instrument, USA). According to the manufacturers, the acidity of the first product (Beverage 1) was linked to its carbonic and phosphoric acid content, while that of the second product (Beverage 2) to carbonic and citric acid. Ethical clearance for this work was provided by the Institutional committee for ethics and research.

-Conditioning of Specimens

The specimens were cleaned in distilled water in an ultrasonic cleaner for 1 minute to remove any debris. Prior to the tests, all the specimens were stored in distilled water for 7 days (8).

-Surface microhardness measurements

The specimens were then blotted dry and repositioned inside the acrylic blocks, centrally beneath the indenter of the digital knoop microhardness tester [Clemex, Model MMT-X7, Matsuzawa Co. Ltd, Japan] to estimate the initial surface microhardness (SMH) in Knoop hardness number (KHN). A 50 gf load was applied through the indenter with a dwell time of 10 seconds. [The initial SMH determination was performed by five indentations of each sample for selection purposes. The mean initial SMH for all enamel samples was calculated and the samples whose mean SMH ranged between 10% of the total mean were included (9).

**Figure 1:**
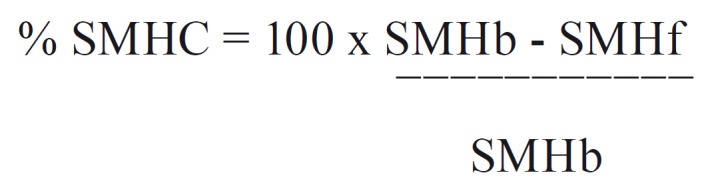
Formula.

The percentage of surface microhardness change for enamel was calculated according to the formula: (Fig. 1).

Where, b = baseline and f = final

-Experimental Design

The study design comprised of three groups: Group 1 had three cycles of a 5-minute immersion of the restorative specimens in the beverages separately, interspaced by 5-minute storage in artificial saliva, repeated once in a week for a month. The artificial saliva was mixed according to the formulation given by Klimek *et al.* (10). The specimens were rinsed in normal saline before and after each immersion. Apart from the exposure time, the restorative specimens were stored in de-ionized water in airtight plastic containers at room temperature and carefully labelled (11). In Group 2, the specimens had three cycles of similar 5-minute immersion in a day, but repeated on a daily basis for a month; while in the Control group, the specimens were stored in de-ionized water all throughout the study period with no exposure to the acidic beverages at all. After 1 month of experimental conditioning in all the groups, the final SMH of the specimens were recorded.

-Statistical analysis

KHN data were subjected to statistical analysis (SPSS version 15.00) at 5% significance level. The students’ T-test and one-way ANOVA were applied for inter-comparisons between the groups.

## Results

A total number of five indentations were made on a surface per sample and its mean was calculated. The mean value thus obtained was considered as the microhardness of the surface tested.  Table 1,  Table 2 show the mean Knoop hardness number (KHN) of five restorative materials following exposure to the beverages in the 3 groups tested.

**Table 1:**
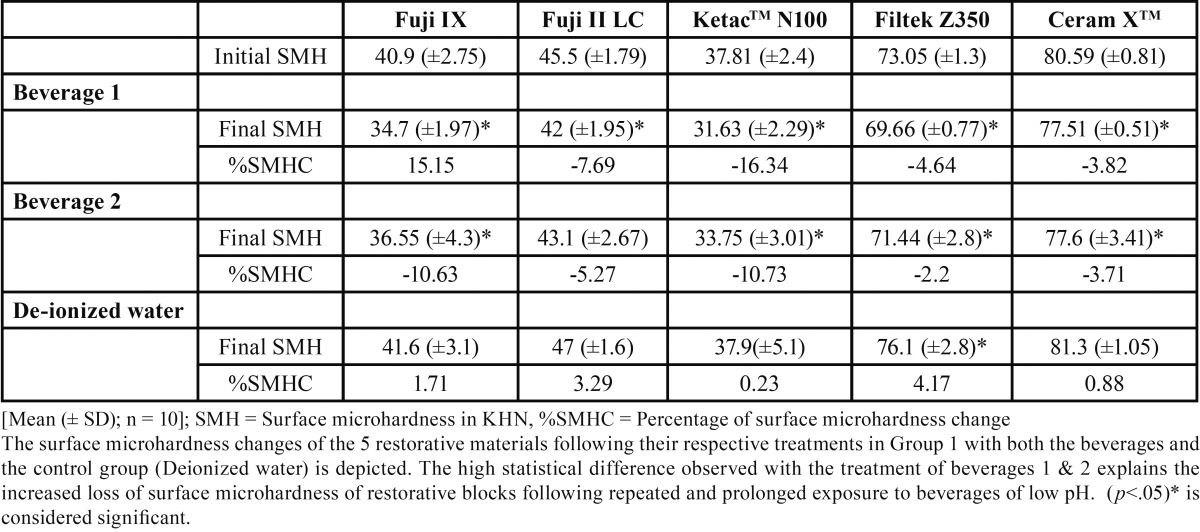
Treatment of the restorative materials in Group 1.

**Table 2:**
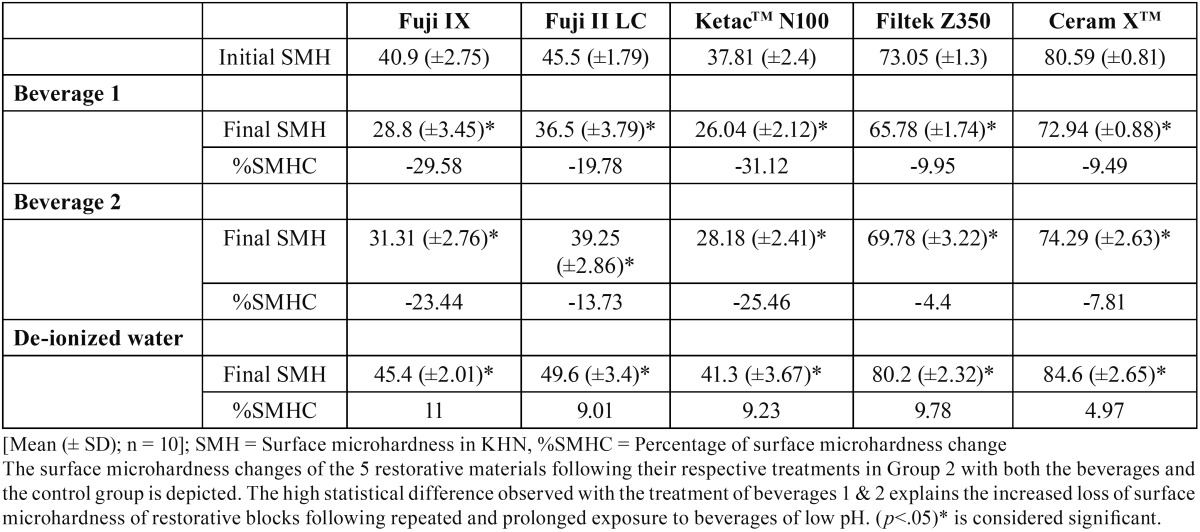
Treatment of the restorative materials in Group 2.

The 5 restorative materials were compared from their initial mean surface microhardness to their final values in all the groups using the paired student t-test. A high statistical difference (*p*<.0005) was observed between Group 1 and 2 when treated with both the beverages ( Table 1,  Table 2). The high statistical difference observed in these groups self-explains the increased loss of surface microhardness of restorative blocks following repeated and prolonged exposure to beverages of low pH. However in the control group, the restorative specimens showed an inverse effect with SMH, gradually increasing over the 30 days storage period ( Table 1,  Table 2) (*p*<.05).

Inter-comparisons of KHN values among the 5 restorative materials between Group 1 and 2 showed an increased SHM reduction with the samples treated with beverage 1 than beverage 2 (*p*<.05). The present data thus affirms that total acid content or pH of a beverage directly attributes to the loss of hardness among the restorative materials tested.

The one-way ANOVA inter-compared the SMH changes between the five restorative samples. Though the surface microhardness loss was observed with all the restorative specimens in Group 1 and 2, the maximum reduction was noticed with the KetacTM N100 followed by the Fuji II restorative (*p*<.0005).

## Discussion

Restorative materials in the oral cavity are frequently exposed to variables including temperature changes and acid-base conditions (6). Despite the mouth being the ultimate testing environment for predicting the behavior of restorations due to the complexity and diversity of intra-oral conditions, *in-vitro* models are essential for providing an insight into the fundamental mechanisms of biodegradation (11). It is known that, during consumption, food or drink only comes in brief contact with tooth surfaces/restorations before it is washed away by saliva (12). This study aimed to assess the surface microhardness changes of restorative materials following acidic challenges, by stimulating an oral behavior in an *in-vitro* situation. The effects of attrition from chewing habits however weren’t assessed in the current study, the oral cavity being a complex environment (6).

Mostly all soft drinks available in the market today have phosphoric acid, which gives a peculiar tangy taste and is known for its preservative property. The contents in soft drinks absolutely affect the integrity of dental enamel and are responsible for its erosion (13). The cola drink was chosen as an erosive inductor because of its low pH and low calcium and fluoride concentrations (12). Based on the data showing that the pH of oral fluids returned to neutral 1-3 min after one single sip of an acidic beverage (14), the 5 min immersion in each cycle was selected. This method was applied in an attempt to simulate the regular intake of individuals considered at high risk for dental erosion, though it may not exactly reproduce the clinical situation. Once the pH drops to a level below the critical value, salivary flow rate increases and the beverage gets diluted by the saliva (15).

Material storage before pH cycling can affect its behavior. Francisconi *et al.* found the greatest change in hardness of composites to occur within the first seven days of experimental conditioning (11). The initial conditioning period similarly chosen in this study was to allow post-irradiation hardening of the composites and stabilization of the acid-base reaction of glass ionomer cements (16-18).

Previous investigators have shown that the most acidic drinks tend to show the greatest effects on teeth (19). Although the complex nature of the degradation suffered by dental materials and dental hard tissue subsequent to an erosive and cariogenic challenges, it is observed that surface microhardness assesment is an appropriate method to verify small alterations in mineral content after acid demineralization (20). The current study of repeated exposure of carbonated beverages to restorative materials similarly converges on this harmful effect, marked by a decline in the final microhardness from their initial values. The degrading effect of acidity on restorative materials has been a subject of concern for research since long. Lawrence Mair and Joiner A studied the degradation and wear of Glass ionomer following peroxide bleaching and has shown a decrease in hardness (21). Olga Polydorou *et al.* studied the effects of bleaching on the microhardness of composites and also reported a similar decrease (22). Although variables such as titratable acidity and buffering capacity of a beverage have been suggested to be important variables in wear, the present data confirms that the total acid content or pH to be responsible for the loss of hardness in restorative materials. Beverage 1 having a reduced pH than the other product produced more reduction in SMH in the current study (*p*<.05).

The reduction in surface hardness with Fuji II LC restorative may be attributed to the selective attack on the polysalt matrix among the residual particles (23). The polysalt matrix of the set cement results from the formation of contact cation-anion ion pairs or complexes between the carboxylic groups of the polyalkenoic acid and metallic ions. This study also showed Fuji II LC having less erosion compared to Fuji IX. This may be due to the formation of a leachable layer that can inhibit degradation of the material and ability to reduce the acidity of the acidic solutions. The minimal surface alteration in microhardness could also be explained by the type of beverage used for pH cycling and the time of acidic exposure (11). Research also indicates that Fuji II LC may resist acid better than conventional glass ionomer cement (24). Despite the acids adversely affecting the surface integrity of glass–ionomer, this erosive loss of material may be accompanied by an increase in the pH of the acid solution, because of the capability of these materials to buffer external storage media (25). Such buffering effect is likely to be beneficial in protecting the teeth from the occurrence and evolution of dental erosion. However, the preventive additional effect of these materials on enamel subjected to erosion could not be noticed (9).

The deterioration of physical and mechanical properties of Filtek Z350 resin composite could be due to a hydrolytic breakdown of the bond between silane and the filler particles, filler-matrix debonding, or even hydrolytic degradation of the fillers (26). Alternately, it could be due to chemical degradation occurring via hydrolysis. Progressive degradation altered the microstructure of the composite bulk through the formation of pores (18). In the present study, Filtek Z350 resisted acid solution better than did Fuji II LC, which is consistent with the results found in other studies (9,24). Acid could also attack the resin (to a lesser extent), since a reduction in the surface hardness of resin composites soaked in organic acids has been reported, due to softening of Bisp-henol-A-glycidyl methacrylate (Bis-GMA) based polymers, which could result from leaching of diluent agents, such as Triethylene glycol dimethacrylate (TEGDMA) (27). Phosphoric acid could have degraded the zirconia silicate fillers in the Z350 composite in the current study, resulting in a significant decrease in hardness (28). Surface microhardness loss was also observed with CeramXTM restorative, maybe due to the presence of a large number of filler particles responsible for its increased polishing property.

Similar to the findings of Ellakuria *et al.* that reported an exponential rise in surface hardness of restorative materials over a one-year water storage period, an increase in SMH was observed in the control group over a 1-month treatment (29). The resin matrix of composites is known to absorb a small percentage of water, which changes the magnitude of some physical properties. Surface hardness of composites has been reported to be significantly affected by water sorption and the contact time with the aqueous media (30).

It is indeed difficult to isolate restorative materials that can overcome all external challenges and successfully retain their physical, chemical and mechanical properties. Various assessment techniques thus need to be applied to evaluate the degradation of dental materials and the loss of dental hard tissue by erosive challenges, such as microhardness (16,25), surface roughness (26), weight changes (26), compressive, biaxial flexure and shear punch strength (17) and wear (24). Though newer restorative materials serve to fulfill the esthetic concerns of the population, the effect of frequently consumed carbonated beverages on their durability and longevity needs further research. This *in-vitro* study thus might recommend that, in terms of resistance to degradation, resin composite should be the material of choice while restoring teeth affected by erosion. However, the degradation of materials is not the only factor involved in making this choice. Operator decision and an appropriate patient selection should be taken into consideration.

Within the limitations of this study it can be concluded that repeated and long term exposure to acidic beverages potentially affects hardness of esthetic dental restorative materials. The surface microhardness loss was highest with resin modified light cure nano-glass ionomer (KetacTM N100) and the least with visible-light activated direct restorative nanocomposite (Filtek Z350 Composite) upon exposure to the beverages. It was also noticed that the beverage containing phosphoric acid produced increased surface hardness loss in the restorative specimens than those containing citric acid. Thus, preventive advice to public on the consumption of such beverages goes indispensable.
